# Supplementary material for: Deconstruction by C. thermocellum—from microbe mediated to dynamic redistribution of cellulosomes
Source: Life Sci Alliance. 2026 Jan 16;9(3):e202503239. doi: 10.26508/lsa.202503239 (PMC12811415; doi:10.26508/lsa.202503239)
Supplement: Supplementary file 1 [file LSA-2025-03239_Supplemental_Data_1.docx]

**PA-GFP Expression for super high resolution PALM imaging**

The Photoactivated Green fluorescent protein (PA-GFP) was express a plasmid using Q5 High-Fidelity DNA polymerase (New England BioLabs, Ipswich, MA, U.S.A. The codon-optimized CtDoc-GFP expression vector was synthesized by GeneScript (Piscataway, NJ, U.S.A.). pDCYB 209, containing the point mutation (T203H) at amino acid position 203 of the GFP coding sequence as shown in the figure S1A, was generated using overlapping polymerase chain reaction (PCR) (Figure S1) [44]. *E. coli* strain DH5a was used as the transformant.

**Spatial resolution of the super-high resolution microscope**

For conventional microscopy, the lateral resolution is given calculated by the Abbe Equation [23]

$r_{lat}=\frac{0.61\lambda}{N.A.}$ (1)

And the axial resolution is defined as [1]

$r_{ax}=\frac{2n\lambda}{\left( N.A. \right)^{2}}$ (2)

Super-high-resolution techniques enable microscopy to break the diffraction limit by temporally or spatially modulating the excitation or activation of light via the chromophores. Data collected from the super high resolution microscope were generated by iterating the optical images and capturing individual cells in both the log phase and the stationary phase of growth. Each bacterial cell was analyzed with the DBSCAN clustering algorithm by keeping the epsilon constant at a 75-nm radius and iterated through the MNM. Because the various bacterial cells did not necessarily have the same number of clusters, nor did they have the same MNM, the data from the DBSCAN cluster analysis for each bacterium were normalized, and the normalized data were averaged among all other bacterial cells.

Figure S2 shows a normal fluorescent image of bacteria attached to Avicel (Figure S2A). The bacteria are labeled with the AF 647 chromophore attached via an CBM3 antibody located on the cellulosome. The fluorescence is diffuse resulting in the difficulty in distinguishing its structure as compared the super-high-resolution image in figure S2B where fluorescent events can be distinguished resulting in a sharper image. The purple arrow highlights the region used in figure S2C demonstrating the true optical spatial resolution of the STORM/PALM super high-resolution microscope used in this work. There are two circled regions (region C1 and region C2) with the actual measurements shown. Region C1 shows 4 distinct fluorescent chromophores within a total distance of ~130nm.

This highlighted region demonstrates the ability of the system to image below the diffraction barrier of light. These 4 fluorescent events within a low-resolution system would appear as one larger unresolved gaussian fluorescence signal. Region C2 shows the measured spacing of the three fluorescence events to be 60nm, well below the diffraction barrier of light.

Similar spatial resolution is demonstrated with the photoactivated GFP (PA-GFP) as shown in figure S3. Figure S3a is the white light image in conjunction with the fluorescence from the PA-GFP. Figure S3B is the fluorescence from the super high-resolution microscope with the crisp single fluorescent events. Figure S3C shows a selected region of a single bacterium with the PA-GFP attached to a vacant type-1 dockerin site. The region in the circle shows three PA-GPFs separated by ~100nm with a distance end to end of ~280nm. This helps demonstrate that the spatial resolution of both the STORM system (AF647) and the PALM system (PA-GFP) is below the diffraction barrier of light.

**Quantitative vs Qualitative microscopy imaging**

Traditional optical microscopy does not possess the ability to systematically quantify information within the images and the interpretation of the optical microscopy images is more subjective. Figure S4 demonstrates the visual difference of the fluorescence pattern between *C. thermocellum* in log phase (figure S4A) and in stationary phase (figure S4B.) Figure 3 shows 18 individually selected bacterial sells from the over 60 bacterial cells used in the DBSCAN clustering analysis. Visually, there is a difference between the log phase bacterial cells and the stationary bacterial cells. The issue arises in being able to describe the overall distribution pattern of the fluorescence on the surface of the bacterial cell wall. Visually speaking, the log phase bacterial cells (figure S4A) appear to have an increase in fluorescence on the bacterial wall compared to bacterial cells in stationary phase (figure S4B). From this perspective, we can only speculate if there is truly a reduction in the fluorescence and from figure 3B, out of 18 bacterial cells, 60% appear to have reduced fluorescence. Using our approach, we can perform quantitative analysis on everything single bacterial cell and develop a distribution pattern of the total number of cellulosomes on the surface of the bacteria and correlate with the different phases of growth.

Figure S5 also demonstrate the same issue when looking at bacterial cells on a substrate. Figure S5A shows the fluorescence pattern of cellulosomes on both the bacteria and the Avicel during log phase and figure S5B shows the fluorescence of the cellulosomes on both the bacteria and Avicel during stationary phase. Once again, these are selected images taken from the two datasets. The complex nature of the fluorescence distribution during log phase demonstrates the need to implement the DBSCAN analysis as it is difficult to distinguish the boundary between the Avicel and the bacterial cells.
